# The absence of surface D-alanylation, localized on lipoteichoic acid, impacts the *Clostridioides difficile* way of life and antibiotic resistance

**DOI:** 10.3389/fmicb.2023.1267662

**Published:** 2023-10-30

**Authors:** Pierre-Alexandre Lacotte, Sandrine Denis-Quanquin, Eva Chatonnat, Julie Le Bris, David Leparfait, Thierry Lequeux, Isabelle Martin-Verstraete, Thomas Candela

**Affiliations:** ^1^Micalis Institute, Université Paris-Saclay, INRAE AgroParisTech, Jouy-en-Josas, France; ^2^Institut Pasteur, Université Paris Cité, UMR6047 CNRS, Laboratoire Pathogenèse des Bactéries Anaérobies, Paris, France; ^3^Laboratoire de Chimie, UMR5182, ENS Lyon, CNRS, Université Lyon 1, Lyon, France; ^4^Microbial Evolutionary Genomics, Institut Pasteur, CNRS UMR3525, Université Paris Cité, Paris, France; ^5^Normandie Université, Laboratoire de Chimie Moléculaire et Thioorganique LCMT UMR6507, ENSICAEN, UNICAEN, CNRS, Caen, France; ^6^Institut Universitaire de France, Paris, France

**Keywords:** cell wall, polysaccharides, lipoteichoic acid, D-alanylation, CAMPs, antibiotics

## Abstract

**Introduction:**

The *dlt* operon encodes proteins responsible for the esterification of positively charged D-alanine on the wall teichoic acids and lipoteichoic acids of Gram-positive bacteria. This structural modification of the bacterial anionic surface in several species has been described to alter the physicochemical properties of the cell-wall. In addition, it has been linked to reduced sensibilities to cationic antimicrobial peptides and antibiotics.

**Methods:**

We studied the D-alanylation of *Clostridioides difficile* polysaccharides with a complete deletion of the *dltDABC*operon in the 630 strain. To look for D-alanylation location, surface polysaccharides were purified and analyzed by NMR. Properties of the *dltDABC*mutant and the parental strains, were determined for bacterial surface’s hydrophobicity, motility, adhesion, antibiotic resistance.

**Results:**

We first confirmed the role of the *dltDABC*operon in D-alanylation. Then, we established the exclusive esterification of D-alanine on *C. difficile* lipoteichoic acid. Our data also suggest that D-alanylation modifies the cell-wall’s properties, affecting the bacterial surface’s hydrophobicity, motility, adhesion to biotic and abiotic surfaces,and biofilm formation. In addition, our mutant exhibitedincreased sensibilities to antibiotics linked to the membrane, especially bacitracin. A specific inhibitor DLT-1 of DltA reduces the D-alanylation rate in *C. difficile* but the inhibition was not sufficient to decrease the antibiotic resistance against bacitracin and vancomycin.

**Conclusion:**

Our results suggest the D-alanylation of *C. difficile* as an interesting target to tackle *C. difficile* infections.

## 1. Introduction

The cell-wall of Gram-positive bacteria is a complex network of anionic macromolecules involved in their survival and integrity (Neuhaus and Baddiley, 2003). It is composed of peptidoglycan, surface-associated proteins and polysaccharides (Glaser, 1973; Fischer, 1994; Vollmer et al., 2008). Surface glycopolymers, including wall teichoic acids (WTA) and lipoteichoic acids (LTA), are mainly composed of negatively charged glycosyl-phosphate repeat units (Baddiley, 1970). Besides their importance in shaping and ensuring the integrity of the bacteria, the anionic properties of these glycopolymers confer to the cell-wall a crucial role in cell surface adhesion and biofilm formation (Giaouris et al., 2009; Krasowska and Sigler, 2014; Wu et al., 2021). In a complex and highly competitive gut environment, Gram-positive bacteria must also withstand the onslaught of cationic antimicrobial peptides (CAMPs). These peptides are electrostatically drawn to their anionic cell-wall (Nizet, 2006). As a consequence, the modifications of the surface charge modulate the affinity and the resistance to CAMPs (Nizet, 2006). D-alanylation is an esterification of positively charged D-alanine of WTA and/or LTA. D-alanylation is mediated by the *dlt* operon, encoding the DltA, DltB, DltC and DltD proteins required for this surface charge modification (Neuhaus and Baddiley, 2003). D-alanylation proportions on WTA and LTA are highly variable (Fischer et al., 1981). The addition of D-alanine to the cell-wall polysaccharides has been reported to reduce the sensitivity of Gram-positive bacteria to CAMPs, bacteriolytic enzymes and antibiotics (Fischer et al., 1981; Perego et al., 1995; May et al., 2005; Fisher et al., 2006; Saar-Dover et al., 2012; Lund et al., 2016). Consequently, the specific inhibition of D-alanylation has been proposed as a suitable strategy to increase susceptibility to CAMPs and antibiotics. To that aim, a suicide inhibitor of the protein DltA, the {5’-O-[N-(D-alanyl)-sulfamoyl]-adenosine} named DLT-1, described in *Bacillus subtilis* by May et al. (2005) has been successfully used in *Enterococci* and *Staphylococcus aureus* (Coupri et al., 2019, 2021).

*Clostridioides difficile* is a Gram-positive anaerobe spore forming rod-shape bacterium responsible for increasingly frequent and severe infections (Colomb-Cotinat et al., 2019; Guh et al., 2020). *Clostridioides difficile* infections (CDI) are the most common antibiotic-associated intestinal infections in adults and represent a major public health threat (Colomb-Cotinat et al., 2019; Guh et al., 2020). Vegetative cells can colonize the dysbiotic colon after the dissemination of *C. difficile* spores and their germination in the small intestine (Buddle and Fagan, 2022). The alteration of the gastrointestinal tract microbiota is commonly caused by certain antibiotic families, such as cephalosporins, clindamycin or fluoroquinolones (Modi et al., 2014). In the gut, *C. difficile* still needs to withstand the host defenses, such as the immune factors and CAMPs (Gutsmann et al., 2001). Antibiotic therapy is the main therapeutic option to treat CDI, vancomycin and fidaxomicin being the first-line antibiotics recommended for the treatment. Metronidazole is no longer recommended as a first-line therapeutic option and is only indicated in non-severe CDI (van Prehn et al., 2021). However, resistance against these three antibiotics has been recently described and might represent a new challenge in the management of CDI (Kuehne et al., 2018; Boekhoud et al., 2020; Shen et al., 2020).

Three different cell wall-associated polysaccharides have been described in *C. difficile* (Anwar and Vedantam, 2022). The WTA polysaccharide type-I (PSI) and the WTA polysaccharide type-II (PSII) are glycosyl phosphate polymers directly linked to the peptidoglycan (Ganeshapillai et al., 2008). The third polysaccharide from *C. difficile* surface is the LTA, a diacylglycerol N-acetylglucosamine polymer (Reid et al., 2012). A *dltDABC* operon is present. In response to CAMPs and lysozyme, this operon is expressed under the control of the sigma factor σ^V^ (Woods et al., 2016). In addition, surface D-alanylation has been reported to protect *C. difficile* against a few CAMPs and lysozyme and participates in vancomycin sensitivity (McBride and Sonenshein, 2011). However, the location of D-alanylation on the polysaccharides (WTA and/or LTA) remained unknown and the impact of surface D-alanylation on its physiology is still unclear. In this work, we determine the precise location of D-alanylation at the bacterial surface of *C. difficile*. Furthermore, we observed that D-alanylation is involved in the physicochemical properties of *C. difficile* cell-wall and we evaluated the pharmacological inactivation of D-alanylation as a potential target against antibiotic resistance in *C. difficile*.

## 2. Results

### 2.1. Deletion of the *dltDABC* operon impacts cell-wall D-alanine quantities

**Figure 1:**
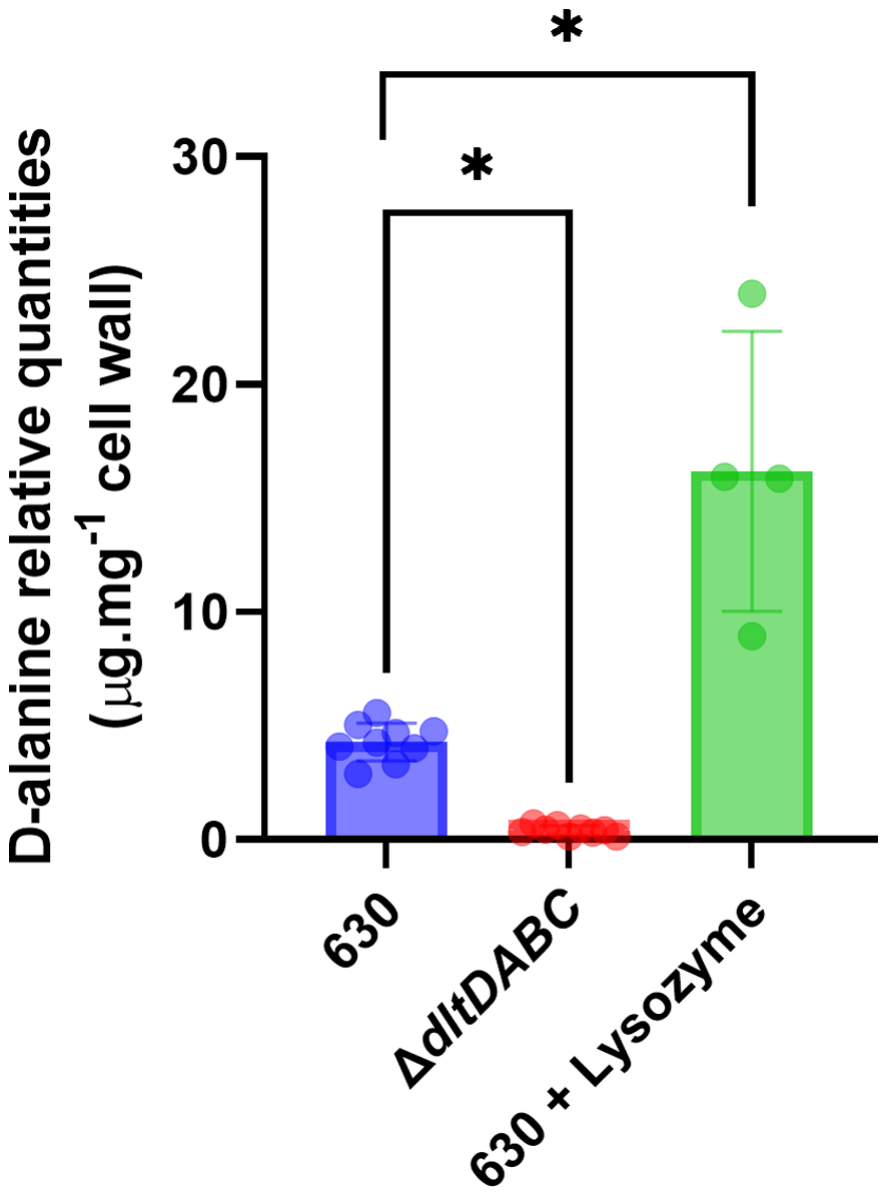
Cell wall D-alanylation in the Δ*dltDABC* mutant and the wild-type strain. We quantified esterified D-alanine in the cell wall of the 630 strain (blue bar), the Δ*dltDABC* mutant (red bar) and the 630 strain in the presence of 600 μg.mL^−1^ of lysozyme (green bar). Results represent the means of six biological replicates. Statistical analysis was performed using an ANOVA test followed by a Tukey test (Asterisks *, *p* < 0.05).

To study the role of D-alanylation in *C. difficile* physiology and antibiotic resistance, we deleted the *dltDABC* operon by allelic exchange in the 630 strain. This deletion was confirmed by PCR (Supplementary Figure S1). The DNA of the mutant was sequenced to verify the genetic construct. The 630 strain and the Δ*dltDABC* mutant grew similarly in TY broth (Supplementary Figure S1) indicating that the inactivation of the *dltDABC* operon did not affect *C. difficile* growth. To further confirm the implication of the *dltDABC* operon in D-alanine incorporation at the surface, we performed a quantification assay of esterized D-alanine. As shown in Figure 1 and Supplementary Figure S2, a very low quantity of D-alanine in the cell-wall of the Δ*dltDABC* mutant induced or not with lysozyme (0.4 μg.mg^−1^) was detected with a 10-fold decrease compared with the 630 strain not induced with lysozyme. We also observed a 4-fold increase of D-alanylation when 630 strain was grown in the presence of lysozyme, in agreement with the induction of expression of the *dlt* operon by lysozyme (Woods et al., 2016).

### 2.2. Location of the D-alanylation in *Clostridioides difficile*

**Figure 2:**
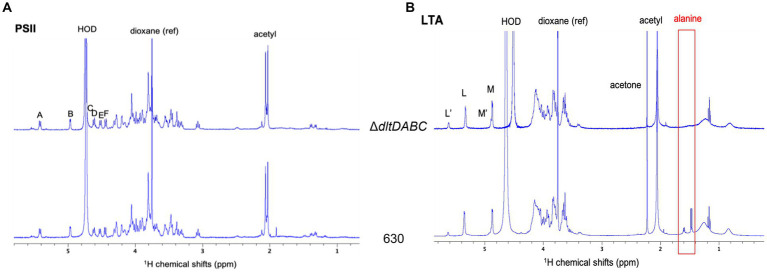
H^1^ NMR analysis of LTA and PSII. ^1^H NMR spectra of LTA **(A)** and PSII **(B)** from Δ*dltDABC* (above) and 630 (below) strains. The signals from the anomeric protons are labeled L, M, L’ and M’. Both samples were solubilized in deuterated water, with 1,4-dioxane as a reference (δ^1^H = 3.75 ppm).

In this study, we intended to identify the specific site of D-alanylation of *C. difficile* glycopolymers. Therefore, we purified LTA and PSII from the Δ*dltDABC* mutant and the parental strain. LTA and PSII samples were characterized by NMR, using ^1^H and ^31^P 1D spectra as well as 2D COSY, TOCSY and ^13^C-^1^H HSQC experiments (Figure 2; Supplementary Figures S3–S5). The proton and carbon chemical shifts are in good agreement with the NMR data previously observed for PSII and o-deacylated LTA (Reid et al., 2012). Thus, LTA seems to be a repetition of a dimer consisting of two N-acetylglucosamine (GlcpNAc) residues (L and M) connected by an a (1-3) linkage. The repeating units seem connected by a 6–6 phosphodiester bridge (6-P-6) between C_6_ of residues L and M, as observed on a ^31^P-^1^H HMBC experiment (Supplementary Figure S4). Both residues are acetylated on C_2_ and M seems to bear a glyceric acid on C_1_. The terminal residues L_t_ and M_t_ are not observed, probably due to sensitivity issues. 15% of the L residues seem to be N-glucosamine (GlcpN), with the substitution of the N-acetyl group at C_2_ for N. This corresponds to a set of signals labeled as L’, and M’ for the other residue in the modified units. Two signals at 1.59 and 1.47 ppm are observed for the 630 strain, which are assigned to CH_3_ from alanine groups, as confirmed by COSY and ^13^C-^1^H HSQC spectra (Supplementary Figure S3). In addition, a DOSY NMR experiment was acquired to determine if the signals from D-alanine are connected to the carbohydrate chain (Supplementary Figure S5). The signal at 1.47 ppm belongs to a small fast diffusing molecule (diffusion coefficient D = 700 μm^2^.s^−1^), probably free alanine. The other signal diffuses at the same slow rate as all other signals from the LTA, with a diffusion coefficient D′ = 22 μm^2^.s^−1^. This shows that the LTA from the 630 strain is substituted by an alanine, on almost 15% of the residues as suggested by the intensity of the CH_3_ signal. Furthermore, the slow diffusion coefficient suggests a molecular mass of 171 ± 12 kDa for the LTA (Augé et al., 2009). ^1^H NMR analysis of PSII from the 630 strain or the ∆*dltDABC* mutant did not show any signal that could be assigned to D-alanine, at 1.59 or 1.47 ppm (Figure 2), suggesting that PSII is not D-alanylated. Altogether, our results suggest that D-alanine esterification is exclusive to the LTA of *C. difficile*.

### 2.3. Absence of D-alanylation impairs *Clostridioides difficile* surface properties, hydrophobicity and adhesion capabilities

**Figure 3:**
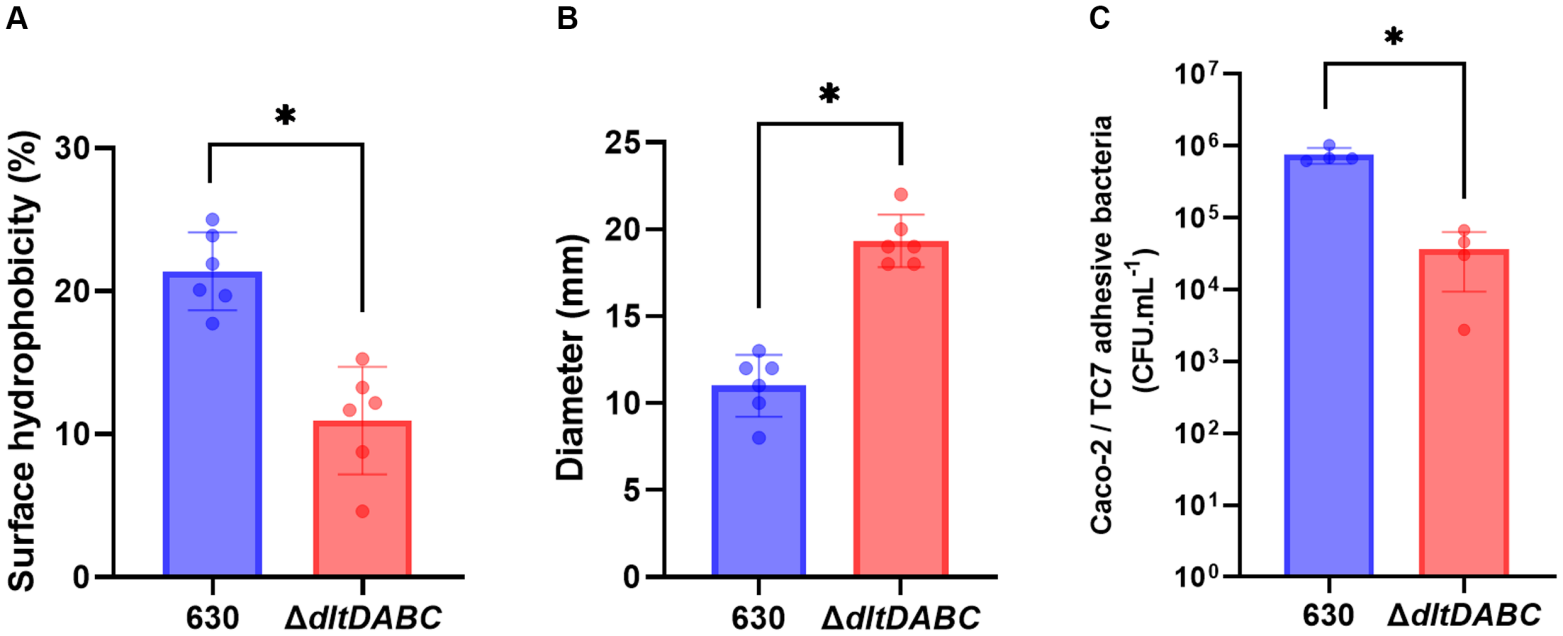
The absence of D-alanylation affects the physical properties of the *C. difficile* surface. 630 strain (blue bars) and the ∆*dltDABC* mutant (red bars) were evaluated for different physical properties. **(A)** The percentage (%) of surface hydrophobicity was reported. **(B)** motility assays with the 630 strain and the ∆*dltDABC* mutant. After growth, the diameter of bacteria representing motility was reported in millimeters (mm). **(C)** *C. difficile* adhesion on TC7 cells was evaluated. The TC7 adhesive bacteria was reported in unit forming colony per mL (CFU.mL^−1^). The results presented are the means of at least five biological replicates. Statistical analysis was performed with a *t* test (Asterisks *, *p* < 0.05).

Modifications of the envelope charge have been reported to be involved in the perturbation of the physicochemical properties of the bacterial cell surface (Giaouris et al., 2009; Nguyen et al., 2011; Wu et al., 2021). Therefore, we investigated the role of D-alanylation of polysaccharides on *C. difficile* surface properties. Surface hydrophobicity was indirectly analyzed by measuring adhesion to the alkane hydrocarbon n-hexadecane. As shown in Figure 3A, the Δ*dltDABC* mutant exhibited a significantly lower affinity for n-hexadecane (12.14% ± 3.57%) than the parental strain (20.88 ± 3.07%). This result suggests that the surface charge modification led to a less hydrophobic surface for the Δ*dltDABC* mutant than the parental strain. No differences in PSII quantities or surface organization in Transmission Electronic Microscopy (TEM) were observed in the Δ*dltDABC* mutant compared to the parental strain (Supplementary Figure S6). Despite the fact that modification of surface charge impacts autolysis in other Firmicutes (Madela and Fixher, n.d.), the deletion of the *dltDABC* operon had no impact on the autolysis of *C. difficile* (Supplementary Figure S7). We also tested the impact of the deletion of the *dltDABC* operon on motility. Using a motility assay on 0.3% agar plates, we observed a significant increase in the motility of the Δ*dltDABC* mutant (19.3 mm ± 1.71) compared to the parental strain (11 mm ± 1.71; Figure 3B). This result was not caused by increased production of FliC in the mutant as observed on Western-Blot using an antibody raised against FliC (Supplementary Figure S8). In some Gram-positive bacteria, changes in the surface hydrophobicity can also impact adhesion (Giaouris et al., 2009; Pantaléon et al., 2018). We then investigated the impact of D-alanylation on adhesion on intestinal Caco-2/TC7 cells. As presented in Figure 3C, the adhesion of the Δ*dltDABC* mutant was significantly lower than that of the parental strain, indicating that the lack of D-alanylation decreases the ability of *C. difficile* to adhere to Caco-2/TC7 cells.

### 2.4. Absence of D-alanylation delays the early steps of adhesion and biofilm formation but enhances the overall biofilm formation properties

**Figure 4:**
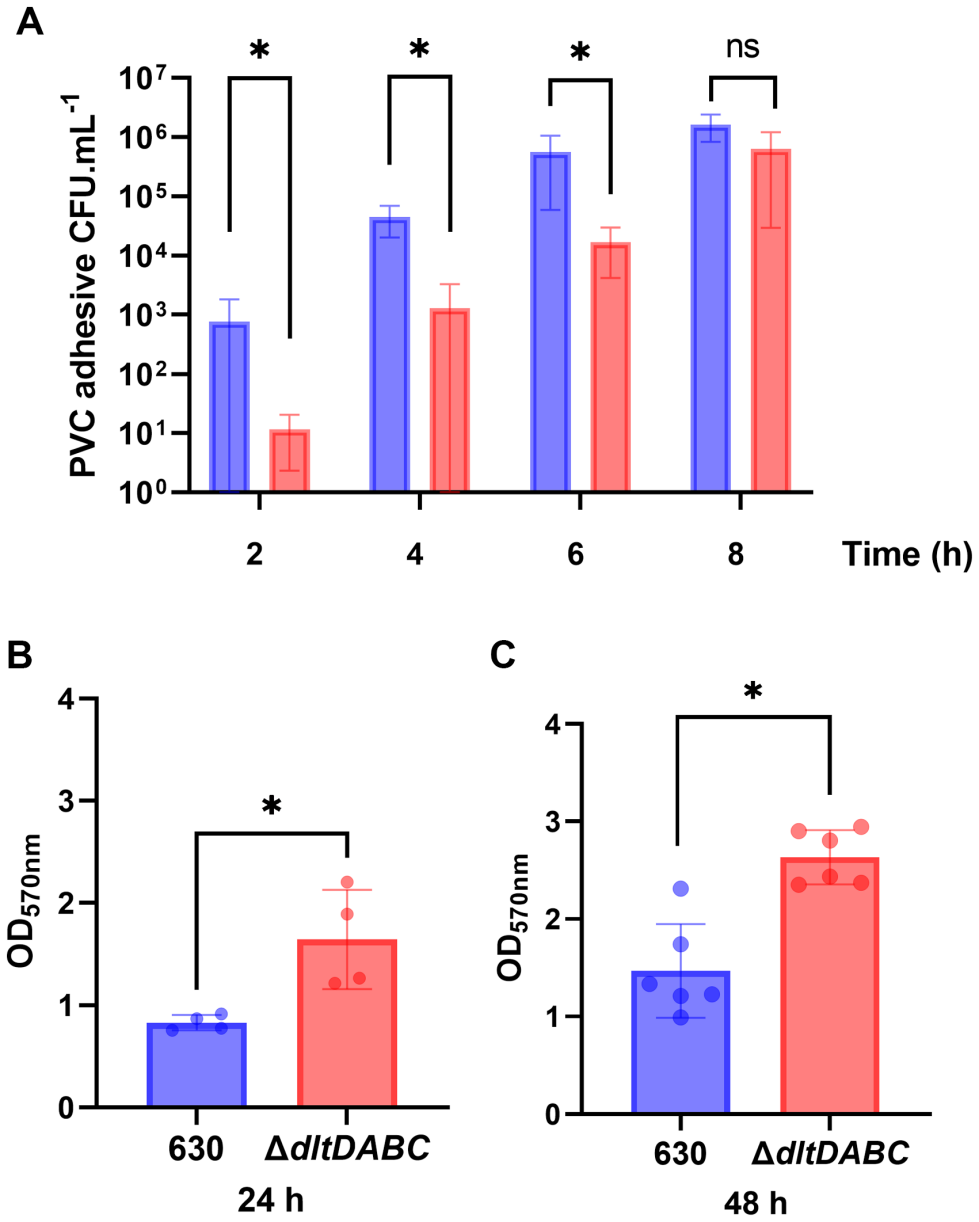
The absence of D-alanylation delays the early steps of biofilm formation but increases the overall biofilm formation properties of *C. difficile*. **(A)** CFU counts per mL of PVC adhesive bacteria for the 630 strain (blue bars) and the Δ*dltDABC* mutant (red bars) during the early biofilm formation steps at 2 h, 4 h, 6 h and 8 h. The results presented are the means of 4 different CFU counts. **(B,C)** Biofilm formation of the 630 strain and the *dltDABC* mutant at 24 h and 48 h. The results presented are the means of 5 biological replicates of crystal-violet staining assays for each strain; OD_570nm_ was reported. **(A–C)** Statistical analysis was performed with a *t* test (Asterisks *, *p* < 0.05); ns, non significant.

Since adhesion and motility are modified in the Δ*dltDABC* mutant, we hypothesized further consequences on other complex biological processes of *C. difficile* such as biofilm formation. Therefore, we first analyzed the early adhesion and biofilm formation steps on a PVC abiotic surface (Figure 4). Means of adhesive bacteria from the Δ*dltDABC* mutant were significantly lower than the parental strain at 2 h (Figure 4A). When bacteria multiply in the early biofilm on the PVC, at 4 h and 6 h, the Δ*dltDABC* mutant was significantly less able to form early biofilms than the 630 strain. By contrast, a difference was not observed after 8 h of early biofilm formation. These results suggest a delay in the phase of adhesion on the abiotic surface and the early steps of biofilm formation up to 6 h for the Δ*dltDABC* mutant. However, the resolution of this delay after 6 h suggests no further consequences on the biofilm formation properties of *C. difficile*. Therefore, we tested the ability of the two strains to form mature biofilms at 24 h and 48 h in BHIS, using a crystal-violet quantification assay. As presented in Figures 4B,C, we observed a significant increase in biofilm formation for the Δ*dltDABC* mutant at 24 h and 48 h compared to the parental strain. Overall, the impact on initial adhesion properties exhibited by the mutant had no consequences on the ability of the Δ*dltDABC* mutant to further form biofilm.

### 2.5. D-alanylation in *Clostridioides difficile* is involved in sensitivity to antibiotics and CAMPs

**Figure 5:**
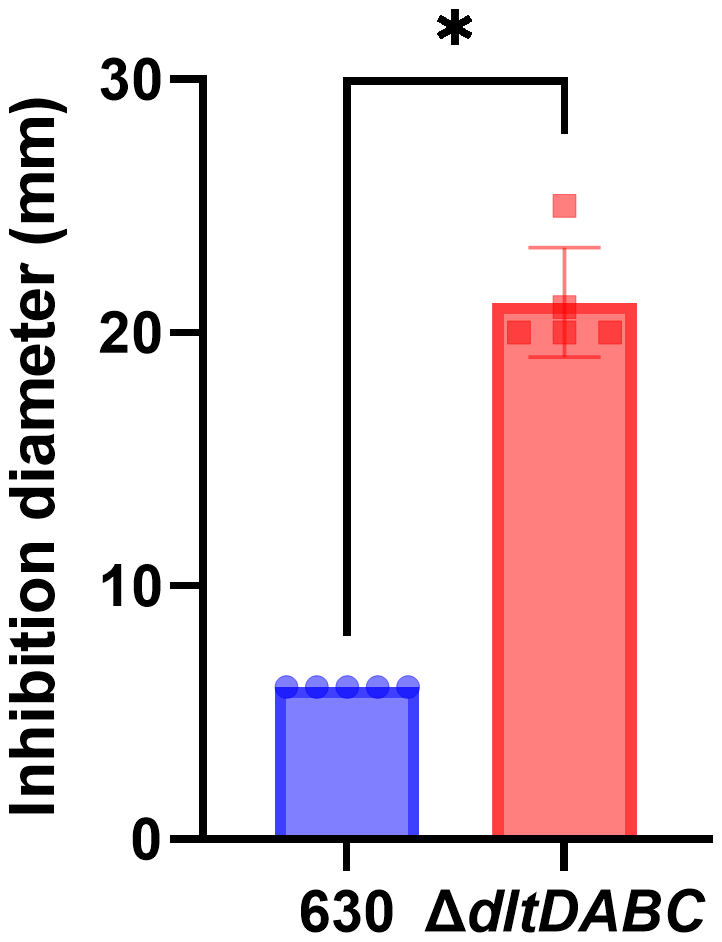
The absence of D-alanylation increases *C. difficile* sensitivity to lysozyme. Sensitivity to lysozyme was assessed by measuring inhibition diameters (in mm) for the 630 strain (blue bar) and the Δ*dltDABC* mutant (red bar). Results represent the means of six biological replicates. Statistical analysis was performed with a Mann–Whitney test (Asterisks *, *p* < 0.05).

As observed using a *dltD* clostron mutant (McBride and Sonenshein; Woods et al.), the esterification of D-alanine at the LTA modulates the resistance to some CAMPs (Nisin, Polymyxin B and Gallidermin) and to lysozyme (McBride and Sonenshein, 2011; Woods et al., 2016). To confirm these results, we tested the susceptibility to lysozyme using an antimicrobial disk assay on Pep-M plates. As shown in Figure 5, we observed a greater size of the zone inhibition for the ∆*dltDABC* mutant (21 mm) compared to the parental strain (7 mm). Taken together, these results confirm the implication of the *dltDABC* operon in lysozyme sensitivity in *C. difficile*, in agreement with previous results (Woods et al., 2016). D-alanylation is a well-known resistance mechanism to antimicrobial compounds and this modification has been previously described in *C. difficile* as involved in the resistance to a few CAMPs (McBride and Sonenshein, 2011). Therefore, we tested the sensitivity of the Δ*dltDABC* mutant to molecules that have an activity close to the bacterial membrane, additional CAMPs (LL37 and nisin) and antibiotics (*i*) targeting specifically the penicillin binding proteins (amoxicillin, imipenem and cefotaxime), (*ii*) targeting the peptidoglycan synthesis (vancomycin, teicoplanin, bacitracin), and (*iii*) targeting indirectly the peptidoglycan synthesis by disturbing the membrane (daptomycin). As presented in Table 1, we observed increased sensitivity of the mutant to bacitracin (>7-fold) and nisin (2-fold), to antibiotics of the glycopeptide family, vancomycin and teicoplanin (2-fold) and to the lipopeptide daptomycin (2-fold). In contrast, we did not observe any modifications in susceptibility to β-lactams, cephalosporins or LL37.

**Table 1:** MICs of the 630 strain and the ∆*dltDABC* mutant or antibiotics targeting cell wall and CAMPs.

	MIC (μg.mL^−1^)	
Compounds	630	Δ*dltDABC*
LL37	16	16
Bacitracin	550	75
Nisin	250	125
Teicoplanin	0.25	0.12
Vancomycin	2	1
Daptomycin	16	8
Amoxicillin	2	2
Imipenem	4	4
Cefotaxime	16	16

MICs were determined using E-Tests. The MICs of LL37, bacitracin, nisin and teicoplanin were determined with the dilution method.

**Figure 6:**
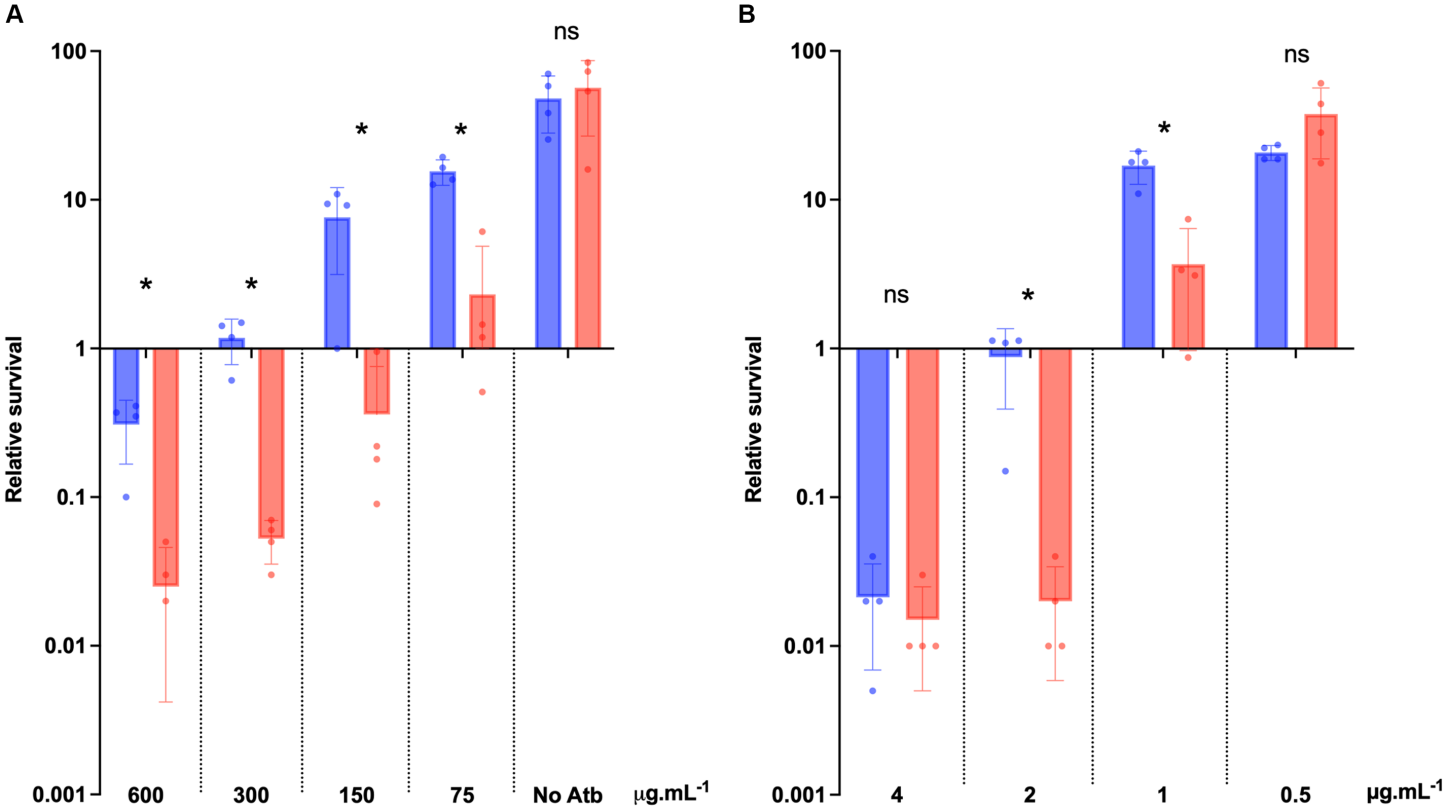
The absence of D-alanylation impairs bacitracin and vancomycin survival. **(A)** Survival assay to bacitracin was performed on *C. difficile* 630 strain (blue bars) and the Δ*dltDABC* mutant (red bars) treated at time 0 (T0) with 600 μg.mL^−1^, 300 μg.mL^−1^, 150 μg.mL^−1^ or 75 μg.mL^−1^ of bacitracin. **(B)** Survival assay to vancomycin was performed on *C. difficile* 630 strain and the Δ*dltDABC* mutant treated at time 0 (T0) with 4 μg.mL^−1^, 2 μg.mL^−1^, 1 μg.mL^−1^ or 0.5 μg.mL^−1^ of vancomycin. Survival was determined after 24 h of incubation (T24) and divided by inoculum at T0 to calculate the relative survival (T24/T0). Results are reported as the mean ± SD from at least four independent experiments. Statistical analysis was performed with a Mann–Whitney test (Asterisks *, *p* < 0.05); ns, non significant.

To precisely determine the increased sensibility of the Δ*dltDABC* mutant to bacitracin and vancomycin, we analyzed the survival of both strains in the presence of those antibiotics at various concentrations including the MICs of the two strains. After 24 h of bacitracin exposure to four different concentrations (MIC, MIC/2, MIC/4 and MIC/8 for 630 strain), we observed a significant decrease in survival to bacitracin of the Δ*dltDABC* mutant in comparison with the wild-type strain at all the concentrations tested (Figure 6A). After 24 h of vancomycin exposure to four different concentrations (2XMIC, MIC, MIC/2 and MIC/4 for 630 strain), we observed a significant decrease in the survival from the Δ*dltDABC* mutant in comparison with the wild-type strain at 2 μg.mL^−1^ and 1 μg.mL^−1^ of vancomycin (Figure 6B). Overall, these results highlight the role of D-alanylation in antibiotics susceptibility in *C. difficile*.

### 2.6. DLT-1 partially inhibits D-alanylation in *Clostridioides difficile* but does not alter its survival to bacitracin and vancomycin

**Figure 7:**
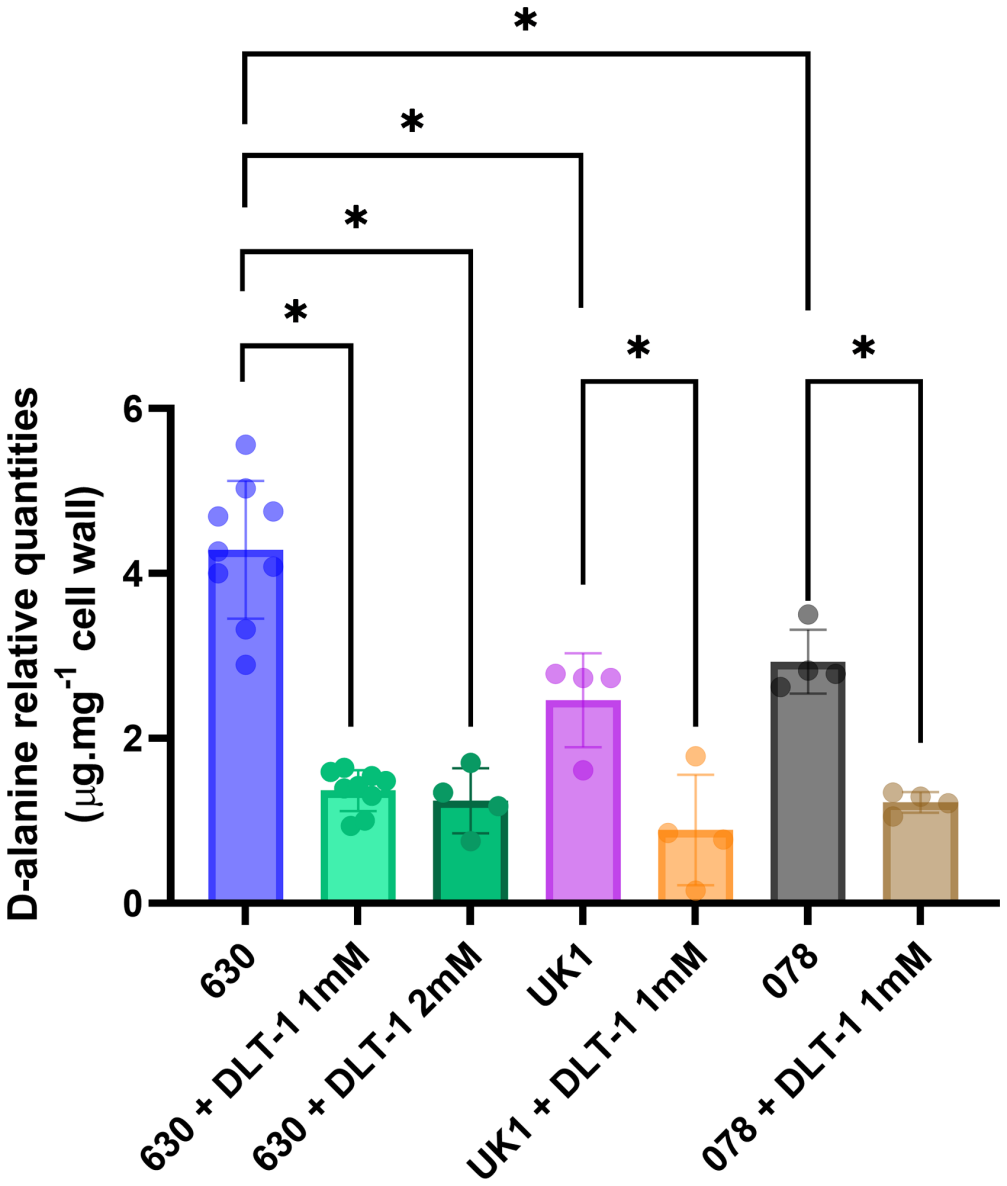
DLT-1 partially inhibits D-alanylation in different *C. difficile* strains. Effect of a DltA specific inhibitor DLT-1 on the esterified D-alanine in the cell walls of the strains 630 (green bars), ribotype 027 (UK1, orange bar) and ribotype 078 (brown bar) strains. Results represent the means of five biological replicates. Statistical analysis was performed using an ANOVA test followed by a Tukey test (Asterisks *, *p* < 0.05).

**Figure 8:**
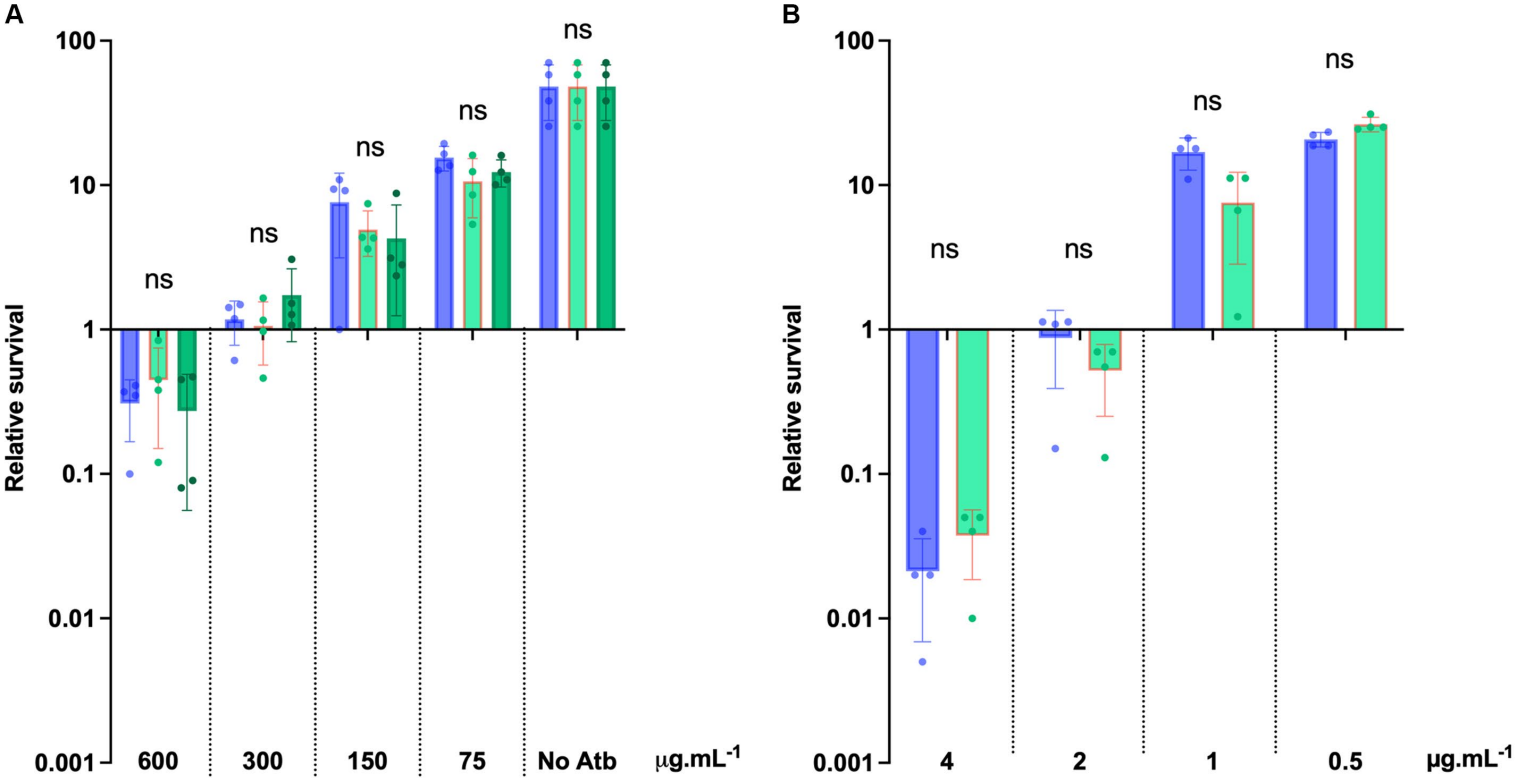
DLT-1 has no effect on bacitracin and vancomycin survival in *C. difficile.* **(A)** Survival assays to bacitracin were performed on *C. difficile* 630 strain treated (green bars) or not (blue bars) by DLT-1 added at time 0 (T0) with 600 μg.mL^−1^, 300 μg.mL^−1^, 150 μg.mL^−1^, 75 μg.mL^−1^ bacitracin. The DLT-1 inhibitor was added at 1 mM (light green) or 2 mM (dark green). **(B)** Survival assays were performed on *C. difficile* 630 strain treated or not by DLT-1 (1 mM) with 4 μg.mL^−1^, 2 μg.mL^−1^, 1 μg.mL^−1^ and 0.5 μg.mL^−1^ vancomycin. **(A,B)** Survival was determined after 24 h of incubation (T24) and divided by inoculum at T0 to calculate the relative survival (T24/T0). Results are reported as the mean ± SD from at least four biological replicates. Statistical analysis was performed with Kruskal Wallis test **(A)** or a Mann–Whitney test **(B)** (ns, non-significant).

To further evaluate the potential of D-alanylation as a druggable inactivation target in *C. difficile*, we investigated the effect of DLT-1, a DltA specific inhibitor first described and tested in *B. subtilis* (May et al., 2005; Coupri et al., 2019). First of all, we assessed the impact of the inhibitor on the quantities of *C. difficile* cell-wall esterified D-alanine quantities. As presented in Figure 7, we tested DLT-1 on 3 different *C. difficile* strains: 630, UK1 (a ribotype 027) and E1 (a 078 ribotype) and quantified the cell-wall esterified D-alanine with and without the inhibitor. The 630 strain possesses a significantly higher quantity of cell-surface D-alanine than the two other tested strains. DLT-1 at 1 mM exhibited a significant decrease in the esterified D-alanine amount detected in cell-wall for all the tested strains, approximatively four-fold (Figure 7). In addition, the effect of the inhibitor was not improved at 2 mM. However, the quantity of D-alanine recovered after DLT-1 inhibition for the 630 strain (2.5 fold reduction in comparison to the 630 strain without treatment) was still higher than the amount detected in the Δ*dltDABC* mutant (10 fold reduction in comparison to the 630 strain without treatment, Figure 1), suggesting only a partial inhibition of D-alanylation of the LTA. We therefore evaluated the impact of DLT-1 on bacitracin and vancomycin survival of the parental strain. The addition of DLT-1 did not modify the profile of survival of the wild-type strain in the presence of an increasing concentration of bacitracin (from 75 μg.mL^−1^ to 600 μg.mL^−1^; Figure 8A) or vancomycin (4 μg.mL^−1^, 2 μg.mL^−1^, 1 μg.mL^−1^ and 0.5 μg.mL^−1^; Figure 8B). Even if DLT-1 significantly reduced the level of D-alanylation of *C. difficile* strain 630, the drastic survival changes observed for the Δ*dltDABC* mutant were not detected in the presence of the DLT-1 inhibitor.

## 3. Materials and methods

### 3.1. Bacterial strains and growth conditions

*C. difficile* strains and plasmids used in this study are presented in Supplementary Table S1. *C. difficile* strains were grown anaerobically (5% H_2_, 5% CO_2_, 90% N_2_) in Brain Heart Infusion broth (BHI), or in a peptone-containing medium (Pep-M; Ng et al., 2013). For solid media, agar was added to a final concentration of 17 g.L^−1^. Cefoxitin (Cfx, 25 μg.mL-1) or thiamphenicol (Tm, 15 μg.mL-1) were added to *C. difficile* cultures when necessary. *Escherichia coli* strains were grown in LB broth. When indicated, ampicillin (100 μg.mL^−1^) or chloramphenicol (15 μg.mL^−1^) was added to the culture medium.

### 3.2. Construction of the *Clostridioides difficile* ∆*dltDABC* mutant, whole genome sequencing and mutant validation

The ∆*dltDABC* knock-out mutant was obtained using an allele exchange method using the inducible toxicity of the CD2517.1 type I toxin (Peltier et al., 2020). Briefly, 1 kb fragments located upstream and downstream of the four genes to be deleted (from *dltD* to *dltC*) were PCR amplified from 630 genomic DNA using primers IMV1286/IMV1287 and IMV1288/IMV1289 (Supplementary Table S2). Purified PCR fragments were then introduced into the pMSR plasmid using the Gibson Assembly^®^ Master Mix (Biolabs). The sequences of the resulting cloning inserts were verified by sequencing. The obtained plasmids, introduced in HB101 (RP4) *E. coli* strain, were transferred by conjugation into the *C. difficile* 630 strain. Transconjugants were selected on BHI plates supplemented with Tm and *C. difficile* selective supplement (SR0096, Oxoid). Isolation of faster growing single-crossover integrants was performed by serial restreaking on BHI plates supplemented with Cfx and Tm. Single-crossover integrants were then restreaked on BHI plates containing 200 ng.mL^−1^ of anhydrotetracycline (ATc) allowing the isolation of double crossover events. After confirmation of plasmid loss (Tm sensitive clones), the presence of the expected deletion was checked by PCR using oligonucleotides IMV1273 and IMV1284. The *dltDABC* mutant and its reference strain 630 were sequenced using Illumina with paired-end 300 bp reads by the diaeresis around Plateforme Microbiologie Mutualisée (P2M – Institut Pasteur). The platform provided filtered pair-end reads, *de novo* assembly and annotation. Deletion of the *dltDABC* operon was confirmed using *breseq* (v0.35.7; Deatherage and Barrick, 2014) with default parameters using filtered reads. This software allows to detect mutation relying on read mapping onto the assembled and annotated reference genomes. The GenBank accession number for the Δ*dltDABC* mutant is JAUPES000000000.

### 3.3. Quantification of D-alanylation

Ester-linked D-alanine quantification assay was performed as previously described (McBride and Sonenshein, 2011) with modifications (Catherwood et al., 2020). Overnight cultures of strains 630 and 630 Δ*dltDABC* in BHI broth were harvested by centrifugation at 5000 g for 15 min. Bacterial pellets were stored at −80°C before analysis. After three washing with 0.1 M MES (pH 6), pellets were resuspended in 1 mL of 0.1 M MES (pH 6) containing 0.2% SDS. Samples were then boiled for 15 min to extract the cell wall. After centrifugation, samples were washed five times in 0.1 M MES (pH 6) and lyophilized for 1 h. Pellets were weighed as total cell wall extract for future comparison. After resuspension of the pellets in 0.5 mL of sodium pyrophosphate (pH 8.3), samples were incubated for 3 h at 60°C to release D-alanine residues. After centrifugation, D-alanine was quantified in the supernatant. The assay reagent contained 2.5 μM of *Rhodotorula gracilis* D-amino acid oxidase, 15 U.mL^−1^ horseradish peroxidase and 0.2 mM of Amplex Red in 0.1 M sodium pyrophosphate (pH 8.3). 500 μL of the sample were mixed with 500 μL of the assay reagent and incubated at 37°C for 15 min. The reaction was stopped by the addition of 0.1% of SDS. After centrifugation, OD_555nm_ was measured for each supernatant. To estimate D-alanine concentration, a standard D-alanine curve was performed. D-alanine relative quantities were calculated by dividing D-alanine concentration by previously weighed total cell-wall extracts.

### 3.4. LTA and polysaccharide II purification and analysis

LTA and PSII were purified as previously described (Cox et al., 2013). *C. difficile* strain 630 was grown in BHI broth supplemented with 0.5 g.L^−1^ cysteine-HCl, 5 g.L^−1^ yeast extract, 1 g.L^−1^ glucose (BHISG) and harvested at an OD_600nm_ of 1. The bacterial cells were centrifugated (8,000 rpm, 4°C, 20 min), killed by adding phenol to 4% and washed with 10 mM phosphate buffered saline, pH 7.4. To isolate the LTA and PS-II, the cells were first extracted in boiling water for 30 min and the resulting solution was separated by low-speed centrifugation. The supernatant was dialyzed against water and lyophilized. Contaminating proteins and nucleic acids were removed from a 5 mg.mL^−1^ aqueous solution of the lyophilized material by precipitation with 15% trichloroacetic acid 16 h at 4°C, low-speed centrifugation followed by dialysis of the supernatant against water. The water-soluble material was separated by anion exchange chromatography on a HiTrap Q column using an H_2_O 1 M NaCl gradient to give PSII. The remaining cells were subjected to extraction with 45% phenol (68°C, 30 min). The water phase was separated from the phenol phase and cell debris by centrifugation. The phenol phase and cell debris were then re-extracted with more water and treated as above. The two water phases were combined and dialyzed against water to eliminate traces of phenol, and then lyophilized. The dried sample was dissolved in water to give a 1–2% solution (w/v) and treated with deoxyribonuclease I (DNase; 0.01 mg.mL^−1^) and ribonuclease (RNase; 0.01 mg.mL^−1^) for 3 h at 37°C, then treated with proteinase K (0.01 mg.mL^−1^) for 3 h. The sample was then dialyzed against water overnight and lyophilized. The resulting LTA containing sample was purified by anion exchange chromatography as above.

For the PSII dot blot, exponential phase cultures were harvested by centrifugation. The supernatant fraction was recovered and precipitated with 10% TCA for 30 min. The supernatant and the total crude cell fractions were treated with 100 μg.mL^−1^ of proteinase K (Sigma) for 1 h at 37°C. Samples were then serially diluted and 5 μL of each dilution were spotted onto an activated polyvinylidene difluoride membrane (PVDF). The membrane was washed in H_2_O, blocked for 15 min in TBST (20 mM Tris–HCl, 150 mM NaCl, 0.05% Tween20, pH 7.5) containing 10% milk, and then washed in 5% milk in TBST for 2 min. After overnight incubation in PSII-LTB rabbit antiserum (1,10,000), the membrane was washed once in TBST with 5% milk, twice in TBST for 5 min, and once in TBST with 5% milk for 10 min. Following incubation with goat anti-rabbit horseradish-peroxidase-conjugated secondary antibody at 1:10,000 dilution for 1 h, the membrane was washed 5 times in TBST for 5 min and revealed using the SuperSignalWest Femto chemiluminescent substrate.

### 3.5. Nuclear magnetic resonance analysis

Lyophilized LTA samples were solubilized in deuterated water. 1,4-dioxane was used as a reference, with δ^1^H = 3.75 ppm, and IUPAC recommendations were followed to reference ^13^C and ^31^P spectra (ref: Pure Appl. Chem., Vol. 80, No. 1, pp. 59–84, 2008). All NMR experiments were performed on a Bruker Avance III 400 MHz spectrometer equipped with a Prodigy Cryoprobe. The temperature was regulated at 298 K and standard Bruker parameter sets were chosen. DOSY experiments used a standard BPPLED pulse sequence with a diffusion time d20 = 200 ms and gradient pulses p30 = 2.5 ms. Diffusion data were analyzed using the DynamicsCenter software implemented in Topspin. The molecular mass of LTA was estimated from the diffusion coefficient using the calculator available.^1^

### 3.6. Test of surface hydrophobicity, motility and biofilm formation

Overnight cultures were diluted in fresh BHI medium to a final OD_600nm_ of 0.05 and incubated at 37°C in anaerobic conditions for 5 h for all assays. Surface hydrophobicity was assessed as the affinity for the apolar solvent n-hexadecane, as previously described (Bellon-Fontaine et al., 1996). The OD_600nm_ of the suspensions (A0) was measured using a spectrophotometer. The bacterial suspension (2.4 mL) was mixed and vortexed for 2 min with 0.4 mL n-hexadecane (Sigma). The mixture was decanted for 15 min to ensure complete separation of the two phases. The absorbance of the water phase (A) was then measured. The percentage of hydrophobic properties was subsequently calculated by the following equation: % hydrophobicity = ((A0-A)/A0)*100.

For motility assays, 5 μL of exponential-growth-phase cultures were cultivated on BHI plates containing 0.3% agar. Plates were incubated for 24 h at 37°C and the zone of motility was then measured. To test biofilm formation, *C. difficile* strains were grown in BHISG broth overnight at 37°C in anaerobic conditions. For biomass quantification and bacterial cell counts, 1 mL of an overnight suspension in BHIS was resuspended at OD_600nm_ 0.05 and cultivated in polystyrene 24-well plates (Costar^®^) for 24 h or 48 h in anaerobic conditions at 37°C. Biofilm biomass was quantified by the classical crystal violet (CV; Acros Organics, United States) staining method. A two-step washing procedure with 1 mL of PBS was applied. Biofilms were air-dried for 10 min at 37°C. 1 mL of 0.2% CV (W/V) was applied to biofilms for 30 min. After the removal of CV, wells were washed twice with 1 mL of PBS. Biofilm biomass was solubilized with an 80:20 ethanol/acetone (V/V) solution, scrapped from the plate and quantified by the measure of OD_570nm_ (V-1200 Spectrophotometer, VWR).

### 3.7. Adhesion assays

Adhesion assays on PVC were performed on 24-well plates (Costar^™^). Overnight cultures were diluted into fresh BHISG broth to a final OD_600nm_ of 0.05. Plates were inoculated with 1 mL of cultures for 2 h, 4 h, 6 h and 8 h. After two wash steps with 1 mL of PBS to remove non-adherent bacteria, adhesive cells were resuspended in 1 mL of PBS. Viable bacteria were then plated on BHISG and cultivated at 37°C for 24 h.

Cell adhesion assays were carried out on Caco-2/TC7 cells. Cells were cultivated to confluence from frozen stock in 24-well plates (Costar^®^) in an MDEM medium. On infection day, Caco-2/TC7 cells were washed once with 1 mL of MDEM medium and incubated in 500 μL of MDEM before infection. Bacteria were grown in BHI broth overnight and diluted at an OD_600nm_ of 0.05 in fresh BHI. After 3 h at 37°C, OD_600nm_ measurements were reported on the 630 strain growth curve to calculate CFU/mL. Strains were then diluted to a final concentration of 2×10^5^ CFU/mL and centrifugated at 7000 rpm for 10 min. Pellets were resuspended in an MDEM medium and 500 μL of bacterial suspension (10^5^ CFU/mL) were added to the Caco-2/TC7 cells. After 1 h of incubation at 37°C in anaerobic conditions, cells were washed twice with 1 mL of PBS. Cells lysis was carried out with 1 mL of Saponin and adherent bacteria were plated on BHI medium, incubated for 48 h at 37°C in anaerobic conditions.

### 3.8. Antimicrobial sensitivity tests

For Lysozyme sensitivity assays, exponential-phase cultures of *C. difficile* strains in Pep-M medium were plated on Pep-M agar plates. Lysozyme (800 μg) was placed on a 6-mm paper disk. The growth inhibition diameter was measured after 24 h of incubation at 37°C.

Strains cultivated overnight were diluted to an OD_600nm_ of 0.05 in fresh BHI medium and incubated at 37°C for 5 h to obtain bacteria in exponential phase before conducting MICs. MICs were determined on BHI plates by E-test (bioMérieux) after 24 h incubation at 37°C. MICs were also determined in liquid culture as follows: 100 μL of BHI were distributed in a 96-well microplate (Bio-Rad, CA, United States). 100 μL of a 4X antibiotic solution were added into the first well and serially diluted to half. After a 100-time dilution of these cultures, 100 μL were distributed in each well of the plates, that were incubated for 24 h at 37°C. The MIC was visually determined as the lowest antibiotic concentration that inhibited bacterial growth.

To quantify survival to antibiotic treatment, strains cultivated overnight were diluted to an OD_600nm_ of 0.05 in fresh BHI medium and incubated at 37°C for 5 h to obtain bacteria in exponential phase. Bacteria were diluted 100-times to obtain approximately 5 10^5^ CFU.mL^−1^ and then distributed on a 96-well microplate (Bio-Rad). Antibiotic solutions were prepared at concentrations 4 times higher than the MIC and serially diluted in half. A DltA inhibitor DLT-1 (May et al., 2005; Coupri et al., 2019) was added when indicated. Samples were collected immediately (T0) to confirm the inoculum size and after 24 h of incubation at 37°C to determine the survival of the different strains by plate counting on BHI agar.

### 3.9. Statistical tests

All tests were performed using Graphpad prism software. For analyses of more than three samples, the normality, using a Shapiro wilk test, and the variance, using a Bartlett test were checked. In case of these hypotheses were confirmed, the parametric test of ANOVA was performed followed by a Tukey test. In case at least one of these hypotheses were not confirmed, the nonparametric test of Kruskal Wallis was performed. For the comparison of two samples, the normality, using a Shapiro wilk test, and the variance, using a Fisher test were checked. In case of these hypotheses were confirmed, the parametric test of *t*-test was performed. In case at least one of these hypotheses were not confirmed, the nonparametric test of Mann Witney was performed.

## 4. Discussion

In this study, we report the specific D-alanylation of *C. difficile* LTA in the presence of DltA, DltB, DltC and DltD proteins. In addition, we showed that the esterification of D-alanine at the cell-wall modifies *C. difficile* physicochemical properties and impacts its motility, adhesion, and biofilm formation. We also confirmed the role of D-alanylation in the sensitivity to CAMPs and antibiotics such as vancomycin and bacitracin. It is notable that the level of D-alanylation of both the UK1 and the 078 strains is reduced approximately 2-fold compared to that of the 630 strain, hinting at either a lowered expression of the *dltDABC* operon or lower quantities of LTA.

In Gram-positive bacteria, D-alanylation proportions are highly variable in wall teichoic acids (WTAs) and LTAs (Fischer et al., 1981). LTAs are generally more susceptible to D-alanylation but the majority of Firmicutes harbor D-alanine esters on both WTAs and LTAs (Mac Arthur and Archibald, 1984; Fischer, 1994). Yet, *C. difficile* D-alanylation sites were still unknown. We did not detect the presence of D-alanine on purified *C. difficile* PSII while esterification of LTA by D-alanine was observed (Figure 2). The D-alanine esters were absent on the LTA of the Δ*dltDABC* mutant. We hereby conclude to a specific esterification of D-alanine on *C. difficile* LTA. The esterification of D-alanine to TA requires the presence of an available hydroxyl function (Perego et al., 1995). PSII does not possess ribitol or glycerol (Ganeshapillai et al., 2008) in agreement with the absence of a detectable D-alanylation. On the contrary, LTA harbors an available glycerol with a hydroxyl function that is the probable target of D-alanylation in *C. difficile.* Furthermore, the presence of alanine can be detected by NMR analysis, whether it is linked to the LTA, or free (Supplementary Figure S5). D-alanine esters are reported to have a fragile chemical link to glycerol and ribitol of bacterial WTA and LTA (Fischer, 1994). This free alanine might be cleaved from the LTA during the stringent purification steps.

In other bacteria, the absence of D-alanylation has been reported to alter the net surface charge and the homeostasis of the cell-wall (Fisher et al., 2006; Madela and Fixher, n.d.). In this study, we observed that the absence of D-alanylation on *C. difficile* LTA modifies the hydrophobicity of the envelope (Figure 3). As expected, the lack of the positively charged D-alanine on LTA reduces the affinity of the cell-wall for apolar solvents such as the n-hexadecane, probably by exposing its anionic components. Such modifications have been described to modify the adhesion properties to abiotic and cellular surfaces in other bacteria (Baddiley, 2000; Gross et al., 2001; Chan et al., 2007; Xia et al., 2010). Here, we report that the absence of D-alanylation in *C. difficile* reduces the adhesion properties of the bacteria to intestinal cells and delays the adhesion to abiotic surfaces like PVC (Figures 3B, 4A). Similar observations have also been previously made in *Listeria monocytogenes*, as the absence of D-alanylation impairs its adhesion capacities to hepatic cells (Abachin et al., 2002) likely by increasing the negative charge of the surface. In *S. aureus*, the lack of D-alanylation also leads to a defect in adhesion to negatively charged surfaces (Gross et al., 2001). Furthermore, Hyyrylainen et al. recently suggested that the D-alanylation of *B. subtilis* LTA was involved in the post-translational folding of surface proteins through the electrostatic affinity to cations Fe^2+^ and Ca^2+^ (Hyyryläinen et al., 2000). In *C. difficile,* cation accumulation in the cell-wall might also alter the folding and activity of adhesins. It is worth noting that the *dltDABC* operon is negatively regulated by the ferric intake regulator in the presence of high iron concentrations (Berges et al., 2018). D-alanylation of LTA might play a role in the exchange of cationic iron at the cell-wall through electrostatic interactions.

The D-alanylation has already been described in *C. difficile* to be involved in the resistance to CAMPs (gallidermin, polymyxin B and nisin) and antibiotics like vancomycin (McBride and Sonenshein, 2011). The modification of the surface charge by the esterification of positively charged D-alanine probably reduces the affinity of the CAMPs for the cell-wall. In this study, we observed on our Δ*dltDABC* mutant an increased sensitivity to nisin but also to antibiotics like bacitracin, vancomycin, teicoplanin and daptomycin (Table 1; Figure 7). Bacitracin binds and inhibits the subtilisin-type proteases in its metal-free form (Stepanov et al., 1981) and the bacterial membrane undecaprenyl-pyrophosphate lipid carrier when complexed with a Zn^2+^, blocking the peptidoglycan synthesis (Storm and Strominger, 1973). The anionic lipopeptide, daptomycin, targets the membrane of the bacteria, and causes a depolarization and a potassium efflux leading to the cell death in a calcium-dependent manner (Silverman et al., 2003). Vancomycin and teicoplanin are antibiotics from the glycopeptide family, targeting the D-alanyl-D-alanine terminus of the lipid II, but they differ in their structure. Indeed, contrary to vancomycin, teicoplanin possesses a hydrophobic side-chain allowing it to anchor directly to the membrane (Zeng et al., 2016). Interestingly, all these antibiotics have a deep interaction or complete binding to the bacterial membrane, but these compounds differ from CAMPs in their structure and mechanisms of action. In addition, we did not observe modifications of susceptibility to the β-lactams families, suggesting that the surface modifications controlled by D-alanylation do not alter peptidoglycan synthesis. The size of LTA is not precisely determined. However, it was suggested that the LTA linked to BSA used as an antigen contains a maximum of 15 units of [−6)-α-D-GlcpNAc-(1–3)-[−P-6]-α-D-GlcpNAc-(1–2)-D-GroA] (Cox et al., 2013). Such an LTA filament may roughly represent up to 97.5 kDa and up to 22 nm in length since glucose has a size of 0.9 nm. Our results suggested that the LTA filament may be even longer representing up to 171 kDa and 38 nm in length. Since the distance between the membrane and the peptidoglycan layer is of 22 nm in *B. subtilis* (Matias and Beveridge, 2005), the LTA of *C. difficile* might be mostly localized in this space. The D-alanylation of LTA could create a steric hindrance at the surface reducing the sensitivity for antibiotics targeting or directly binding membrane.

Recently, May et al. presented a successful inhibition of D-alanylation in *B. subtilis*, using the DLT-1, {5’-O-[N-(D-alanyl)-sulfamoyl]-adenosine}, as a specific inhibitor of DltA. Several studies then proposed a pharmacological inhibition of D-alanylation with DLT-1 as a potential solution for antibiotic resistance in Firmicutes. For example, DLT-1 has been successfully used to increase the susceptibility of *S. aureus* MRSA to imipenem and *Enterococcus faecalis* to β-lactams and antibiotics combinations (Coupri et al., 2019, 2021). Our study is the first to envisage the specific inhibition of D-alanylation in *C. difficile*. We successfully reduced the quantity of D-alanine at the LTA in three *C. difficile* strains using DLT-1 (Figure 8) although the inhibition of D-alanylation was not complete. Indeed, we observe only a four-fold reduction of the esterified D-alanine at the cell-wall, compared with the 10-fold decrease in the Δ*dltDABC* mutant. This suggests either a reduced affinity of the inhibitor for DltA of *C. difficile* compared to the enzyme of other Firmicutes or a low diffusion of DLT-1 through the envelope and the membrane. This might explain why we could not obtain any significant decrease in survival to bacitracin and vancomycin with the DLT-1 inhibitor (Figure 8). However, inhibiting D-alanylation in *C. difficile* still represents an appealing tool to face the challenges of antibiotic resistance in CDI. Our data suggest that the design of specific Dlt inhibitors for *C. difficile* represents an opportunity to impact *C. difficile* way of life and an additional tool for managing CDI.

## Data availability statement

The data presented in the study (Δ*dltDABC* mutant sequence) are deposited in the GenBank repository, accession number JAUPES000000000.

## Ethics statement

Ethical approval was not required for the studies on humans in accordance with the local legislation and institutional requirements because only commercially available established cell lines were used.

## Author contributions

P-AL: Formal analysis, Investigation, Writing – original draft, Visualization. SD-Q: Formal analysis, Investigation, Writing – original draft, Visualization. EC: Investigation, Writing – review & editing. JLB: Formal analysis, Investigation, Writing – original draft. DL: Resources, Writing – review & editing. TL: Resources, Writing – review & editing. IM-V: Funding acquisition, Project administration, Supervision, Conceptualization, Validation, Writing – review & editing. TC: Conceptualization, Funding acquisition, Supervision, Validation, Writing – review & editing.

## Footnotes

^1^http://abcis.cbs.cnrs.fr/htbin-post/MW/MW.py.
