# Supplementary material for: The absence of surface D-alanylation, localized on lipoteichoic acid, impacts the Clostridioides difficile way of life and antibiotic resistance
Source: Front Microbiol. 2023 Oct 30;14:1267662. doi: 10.3389/fmicb.2023.1267662 (PMC10642750; doi:10.3389/fmicb.2023.1267662)
Supplement: Supplementary file 1 [file Data_Sheet_1.DOCX]

Supplementary Material

Absence of surface D-alanylation, localized on lipoteichoic acid, impacts the *Clostridioides difficile* way of life and antibiotic resistance

Pierre-Alexandre Lacotte^1,2^, Sandrine Denis-Quanquin^3^, Eva Chatonnat^2^, Julie Le Bris^4^, David Leparfait^5^, Thierry Lequeux^5^, Isabelle Martin-Verstraete^2,6^, Thomas Candela^1^

**Correspondence:**Corresponding Author: thomas.candela@universite-paris-saclay.fr

# Supplementary Data

The GenBank accession numbers for the Δ*dltDABC* mutant is JAUPES000000000.

We observed two mutations compared to the sequenced genome of 630 strain (1). A point mutation in *prdR* and a small deletion in an intergenic region (CD630_14182 → / ← CD630_14190) in a phage region. These mutations are also present in our reference strain 630.

# Supplementary Figures and Tables

| Strains | Genotypes | origins |
| --- | --- | --- |
| ***E. coli*** |  |  |
| 10-beta | F^-^ *mcr*A D(*mrr-hsd*RMS-*mcr*BC) f80*lac*ZDM15 D*lac*X74 *deo*R, *rec*A1 *ara*D139 D(*ara-leu*)7697 *gal*K *rps*L(StrR) *end*A1 *nup*G | NEB® |
| HB101(RP4) | *supE*44 *aa*14 *galK*2 *lacY*1 ∆(*gpt-proA*) 62 *rpsL*20 (Str^R^)*xyl-5 mtl-1 recA*13 ∆(*mcrC-mrr*) *hsdS*_B_(r_B_^-^m_B_^-^) RP4 (Tra^+^ IncP Ap^R^ Km^R^ Tc^R^) | Laboratory stock |
| ***C. difficile*** |  |  |
| 630*ermR* |  | Laboratory stock |
| CDIPIP1701 | 630 ∆*dltDABC* (CD2854 to CD2851) | This work |
| **plasmids** |  |  |
| pMSR | ACE vector, Cm^R^-Tm^R^ | Peltier et al, Com Biol 2020 |
| pDIA7071 | pMSR ACE ∆*dltDABC* | This work |

Table S1 strains and plasmids

| IMV1286 | TGGTCATGAGATTATCAAAAGGTTAATGGATATAGGAGTGTATTGTA | partI ∆*dltDABC*, 5’ |
| --- | --- | --- |
| IMV1287 | AGAATCAAGAAATTTGTCTAACCCA | partI ∆*dltDABC*, 3’ |
| IMV1288 | TGGGTTAGACAAATTTCTTGATTCTGAGCTTCAACTGATTTAGAGAGA | partII ∆dlt*DABC*, 5’ |
| IMV1289 | ATCGTAGAAATACGGTGTTTTTTTTTAACTTATCCTTCCATTCATTC | partII ∆*DLTDABC*, 3’ |
| IMV1273 | GACATTCATTTTCCTCCTAAATAT | 3’ *dltDABC* Deletion |
| IMV1284 | TAAGGTAAGGTGTATACTTAACCAA | 5’ *dltDABC* Deletion |

Table S2 oligonucleotides used in this study

## Supplementary Figures


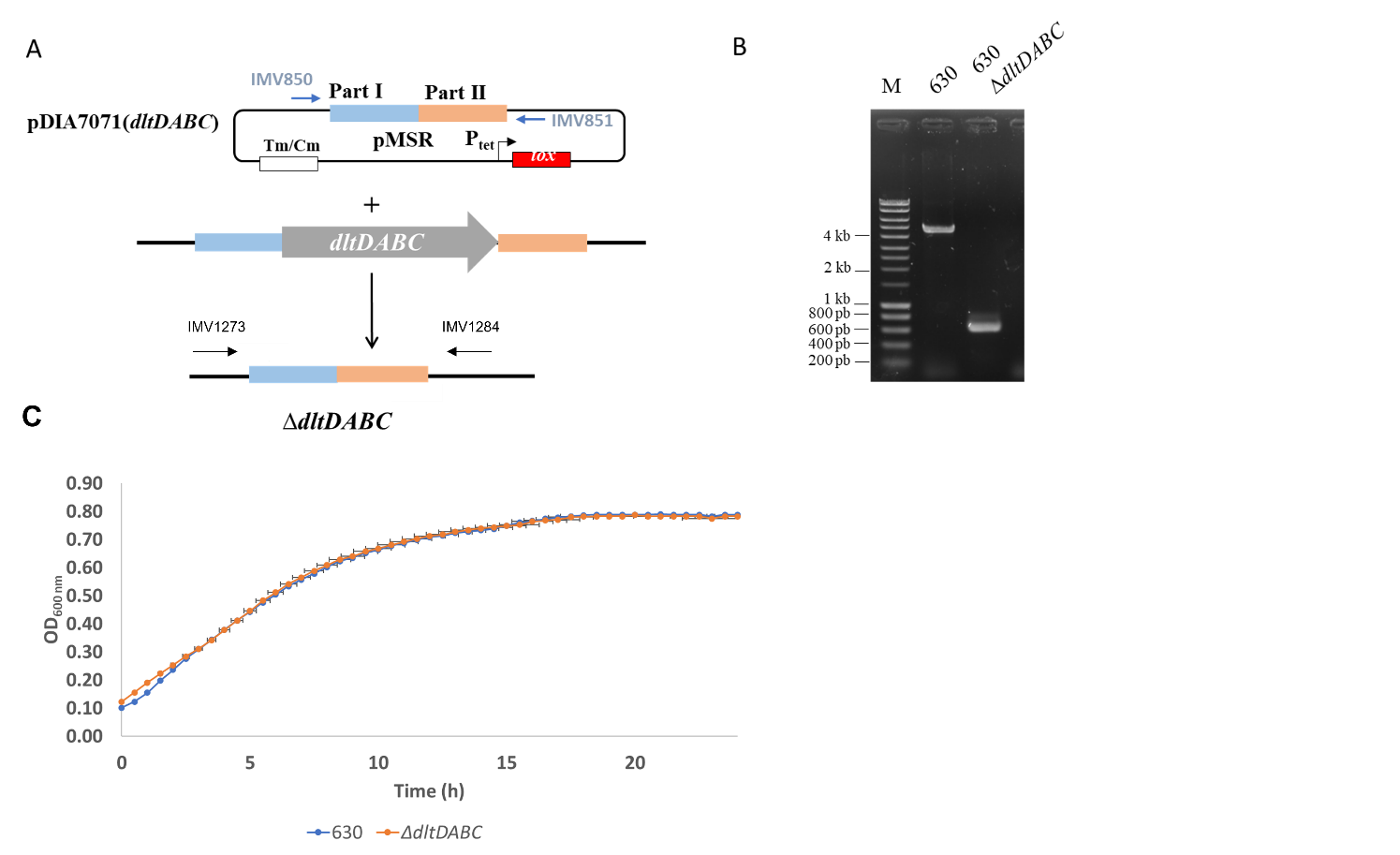


**Fig S1** : **Construction of the Δ*dltDABC* mutant**. (A) Strategy of deletion of the *dlt* operon by allelic chromosomal exchange (ACE). Schematic representation of the *dlt* operon in the 630 and the Δ*dltDABC* mutant obtained by ACE are presented. (B) PCR realized with the oligonucleotides IMV1273 and IMV1284 with chromosomal DNA of the 630 strain (lane 1) or the ∆*dltDABC* mutant (lane 2). (C) Growth curves of the 630 strain and the Δ*dltDABC* mutant. Results presented are the means of five biological replicates.


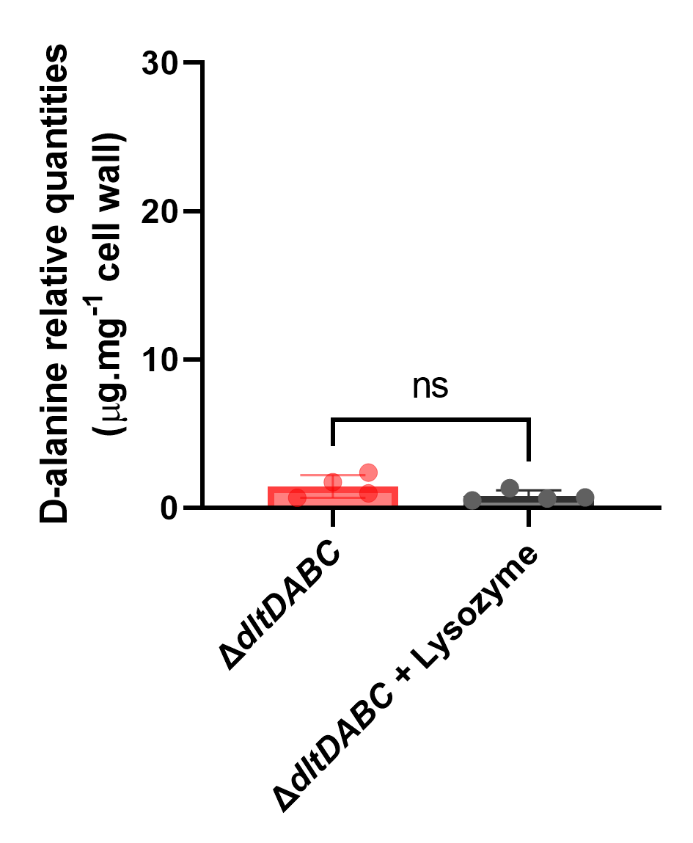


**Figure S2. Cell wall D-alanylation in the Δ*dltDABC* mutant in the presence or absence of lysozyme**

We quantified esterified D-alanine in the cell wall the Δ*dltDABC* mutant strain (red bar) and the Δ*dltDABC* mutant strain in the presence of 600 µg/mL of lysozyme (grey bar). Results represent the means of three biological replicates. Statistical analysis was performed by comparing samples in pairs with *t* tests (ns, non significative)


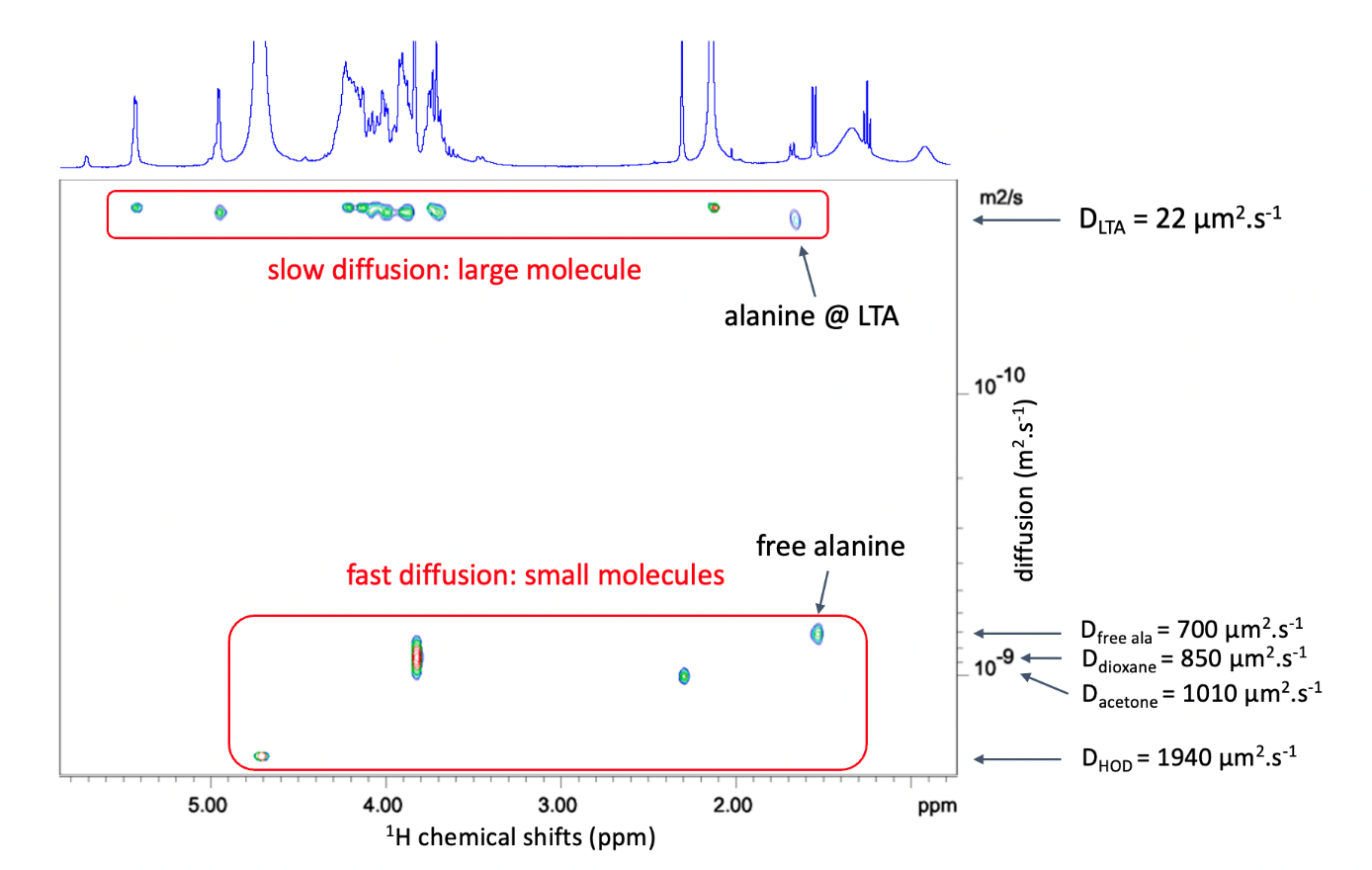


**Supplementary Figure S2: ^13^C-^1^H HSQC of LTA 630**. The signals from the anomeric protons are labelled L, M, L’ and M’.


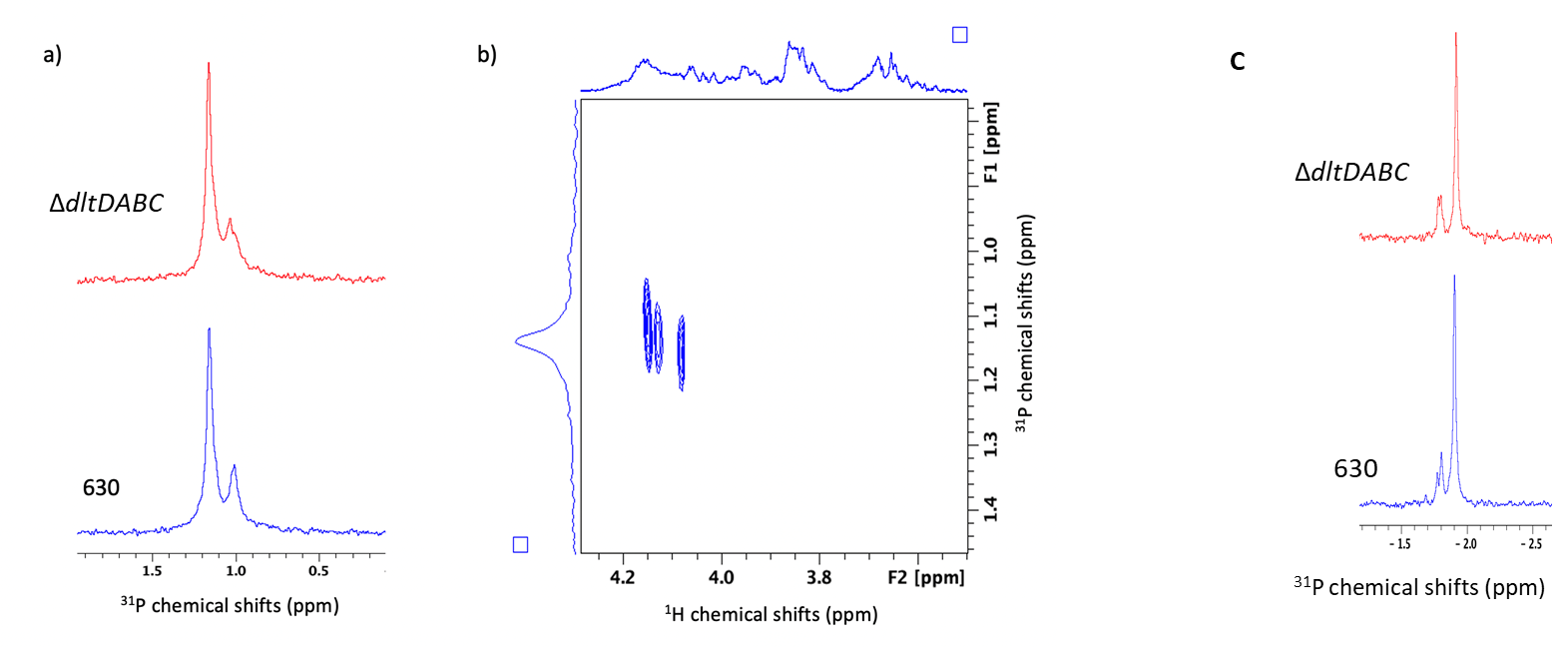
**Supplementary Figure S3**: a) ^31^P NMR spectra of LTA from Δ*dltDABC* (above) and 630 (below) strains. b) ^31^P-^1^H HMBC spectrum of LTA 630 showing correlation with proton 6/6’ from residues L and M. c) ^31^P NMR spectra of PSII from Δ*dltDABC* (above) and 630 (below) strains.

**
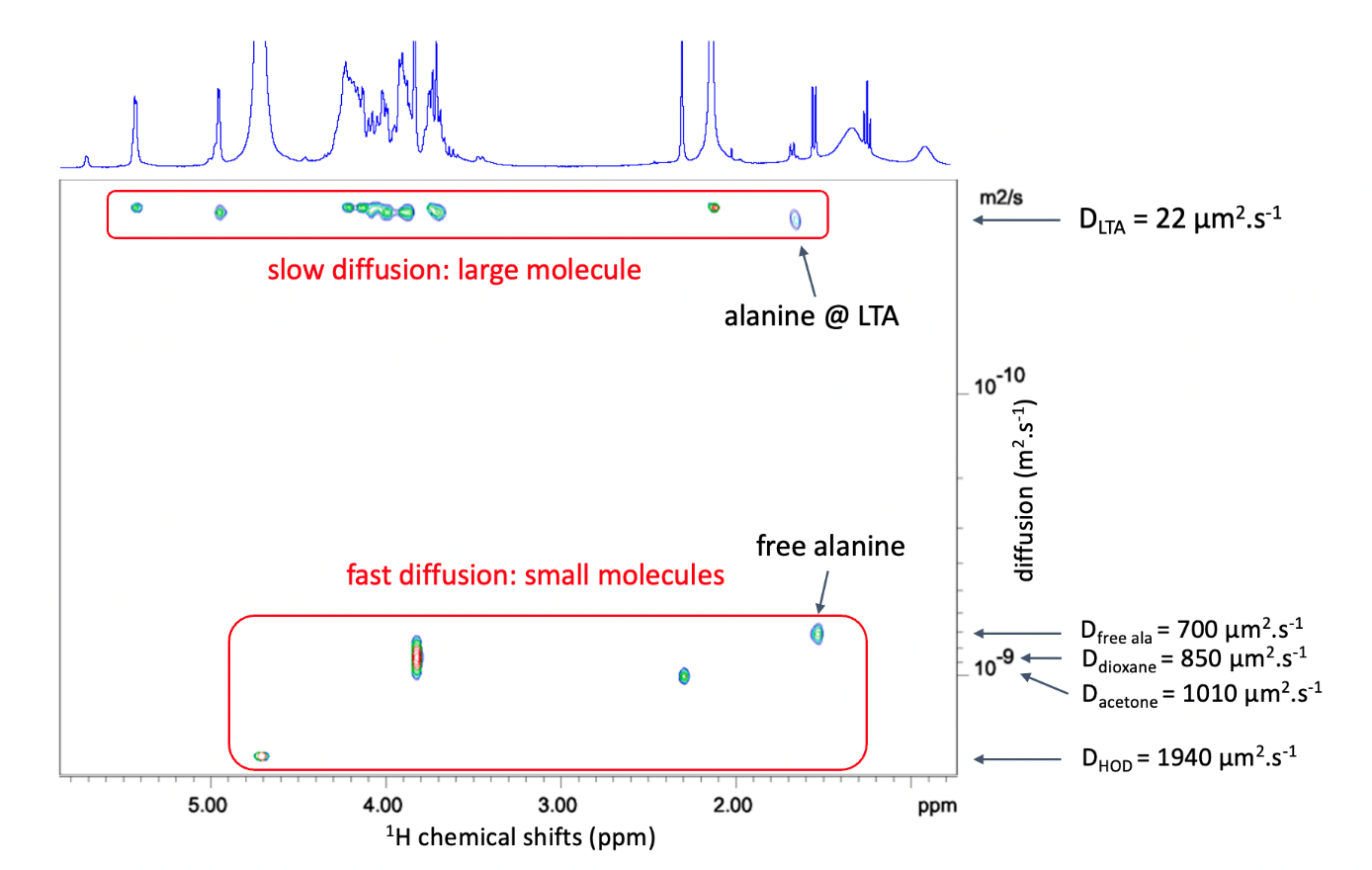
Supplementary Figure S4**: **DOSY spectrum of LTA 630**. The diffusion coefficients D of the main species are given.


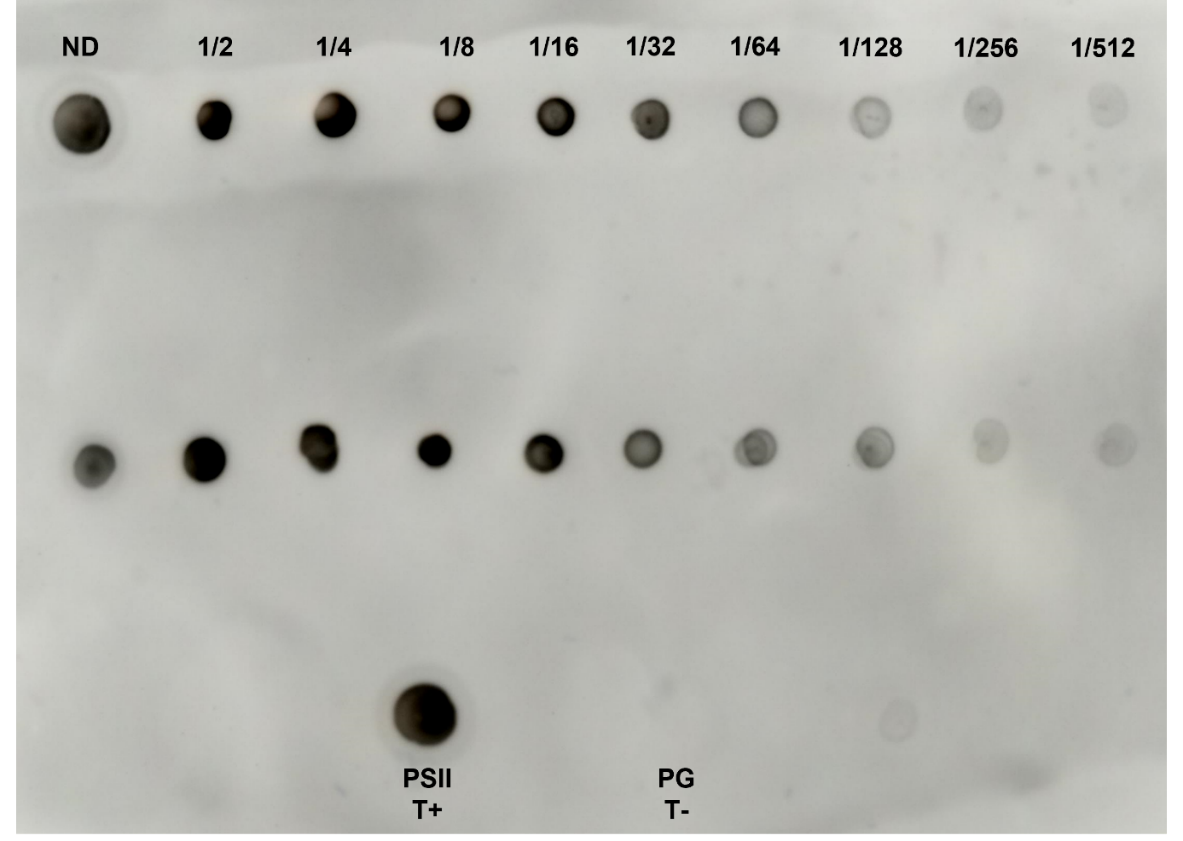


**Supplementary Figure S5 : Dot-Blot of strains Δ*dlt*DABC and 630 using PSII antibodies from Malet-Villemagne *et al.*** (2)


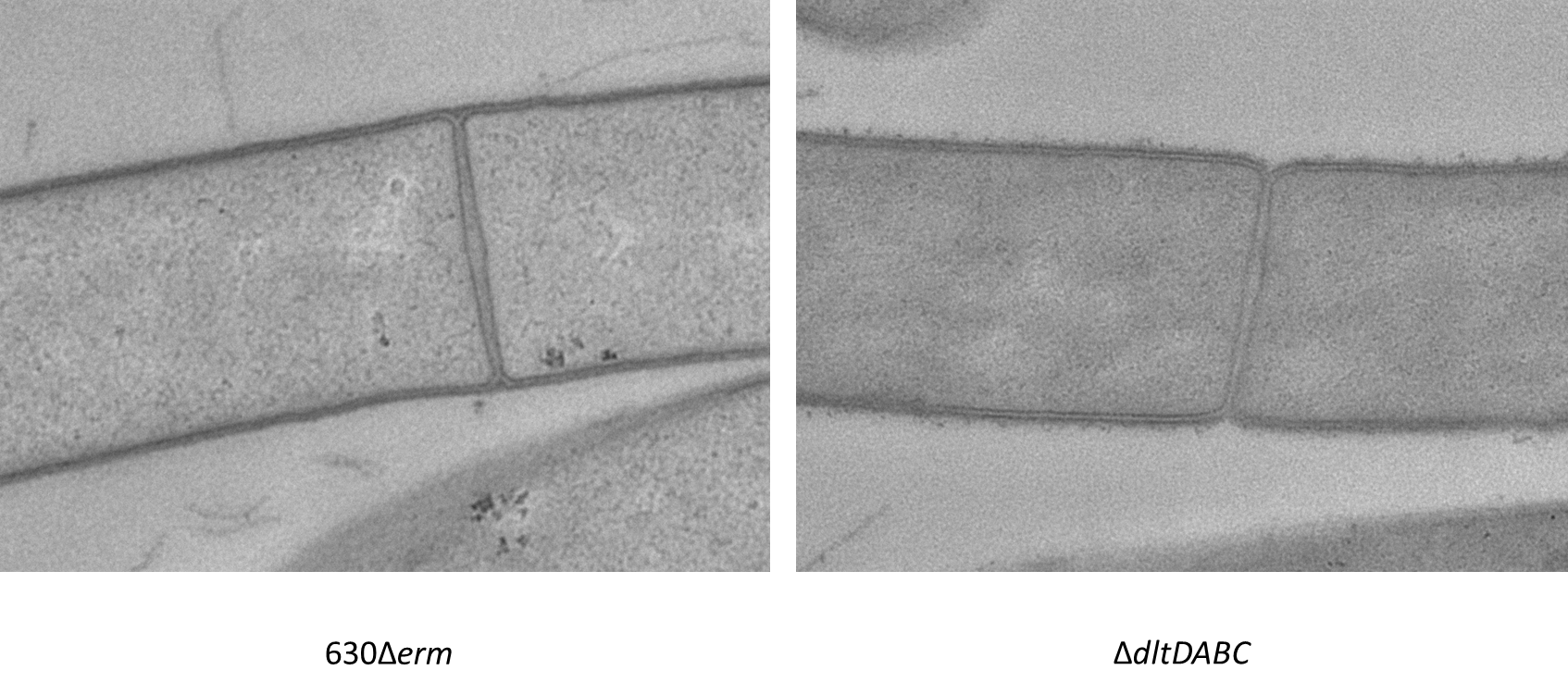

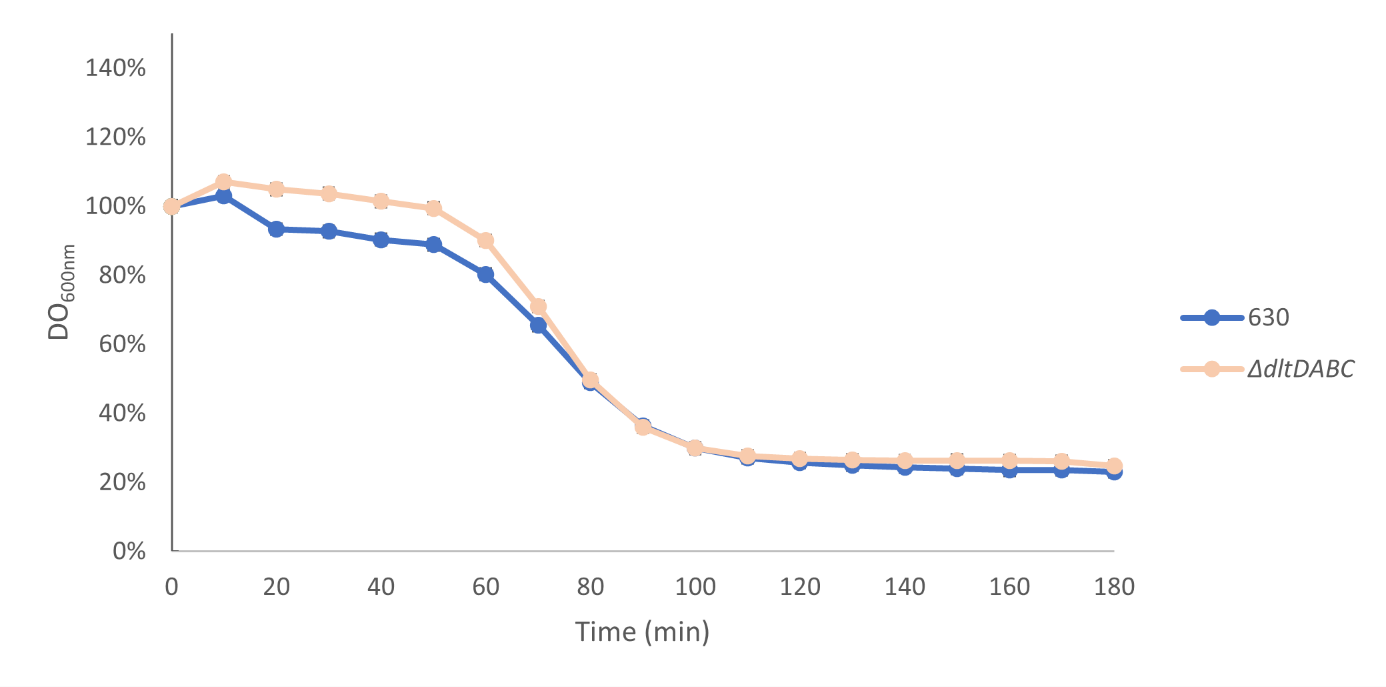
**Supplementary Figure S6**: **Transmission electronic microscope images of the 630 strain and the Δ*dltDABC* mutant.** Samples were prepared as described in Coullon *et al*. (3). Staining and examinations were done by the MIMA2 TEM Platform at INRA, Jouy-en-Josas, France. Grids were examined with a Hitachi HT7700 electron microscope operated at 80 kV, and images were acquired with a charge-coupled device camera (AMT).

**Supplementary Figure S7 : Autolysis profiles of the 630 strain and the ∆*dltDABC* mutant.** Triton X-100-induced-autolysis assay were performed as described by Wydau-Dematteis *et al*. (4), cellular lysis was assessed in the presence of 0.01% Triton X-100.


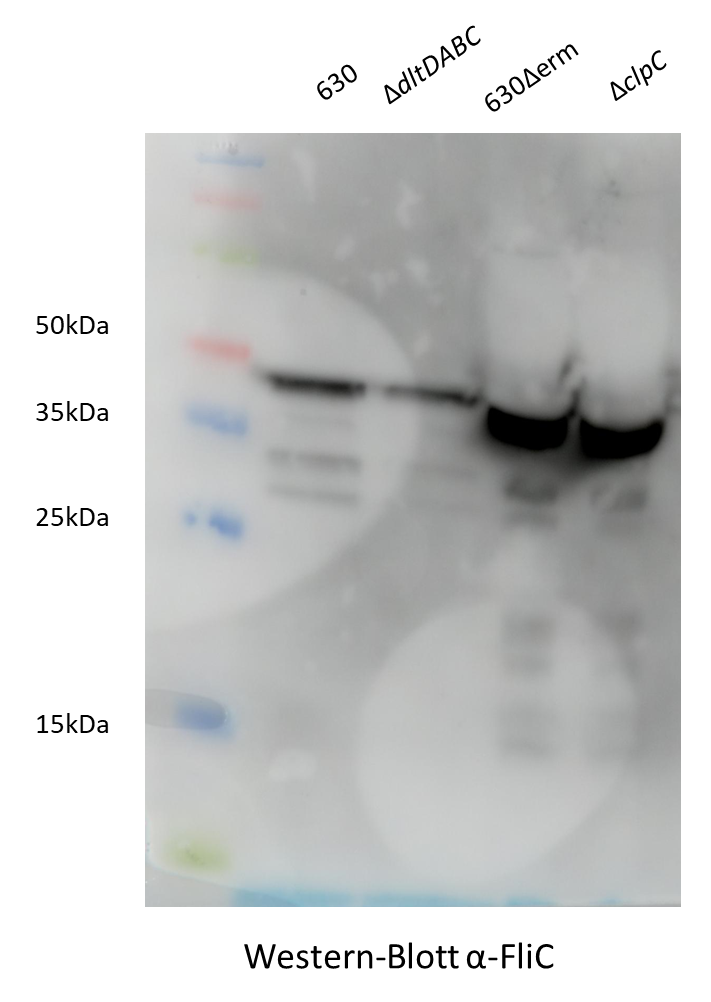
**Figure S8 :** Western-Blot of total protein extracts from the 630 and the Δ*dltDABC* strains using α-FliC antibodies

# References

1. Dannheim H, Riedel T, Neumann-Schaal M, Bunk B, Schober I, Spröer C, et al. Manual curation and reannotation of the genomes of Clostridium difficile 630Δerm and C. difficile 630. Journal of Medical Microbiology. 1 mars 2017;66(3):286‑93.

2. Malet-Villemagne J, Yucheng L, Evanno L, Denis-Quanquin S, Hugonnet JE, Arthur M, et al. Polysaccharide II Surface Anchoring, the Achilles’ Heel of *Clostridioides difficile*. Denes TG, éditeur. Microbiol Spectr. 23 févr 2023;e04227-22.

3. Coullon H, Rifflet A, Wheeler R, Janoir C, Boneca IG, Candela T. N-Deacetylases required for muramic-δ-lactam production are involved in *Clostridium difficile* sporulation, germination, and heat resistance. Journal of Biological Chemistry. nov 2018;293(47):18040‑54.

4. Wydau-Dematteis S, El Meouche I, Courtin P, Hamiot A, Lai-Kuen R, Saubaméa B, et al. Cwp19 Is a Novel Lytic Transglycosylase Involved in Stationary-Phase Autolysis Resulting in Toxin Release in *Clostridium difficile*. Biswas I, éditeur. mBio. 5 juill 2018;9(3):e00648-18.
